# A Brief Review of Paradigm Shifts in Prevention of Alzheimer’s Disease: From Cognitive Reserve to Precision Medicine

**DOI:** 10.3389/fpsyt.2019.00786

**Published:** 2019-10-31

**Authors:** Changtae Hahn, Chang Uk Lee

**Affiliations:** ^1^Department of Psychiatry, Deajeon Saint Mary’s Hospital, College of Medicine, The Catholic University of Korea, Seoul, South Korea; ^2^Department of Psychiatry, Seoul Saint Mary’s Hospital, College of Medicine, The Catholic University of Korea, Seoul, South Korea

**Keywords:** Alzheimer’s disease, cognitive reserve, precision medicine, prevention, aging, biomarkers

## Abstract

Alzheimer’s disease (AD) and related dementias can be an enormous economic burden for taxpayers, patients, their families, medical systems, and society as a whole. Since disease-modifying treatments have failed, several studies have instead focused on a paradigm shift for preventing and treating AD. A higher cognitive reserve (e.g., greater education, occupational attainment, or more leisure activities) is associated with protection against disease-related cognitive decline. Precision medicine aims to optimize the effectiveness of disease prevention and treatment by considering specific biological components of individuals. We suggest that research into cognitive reserve and precision medicine could be a key to overcoming the limitations of traditional approaches to the prevention and treatment of AD.

## Introduction

Dementia is a broad spectrum of neurodegenerative diseases that are characterized by cognitive decline and have a negative impact on daily functions without an acute change of consciousness. In particular, Alzheimer’s disease (AD) refers to a type of slowly progressive dementia that is associated with significant memory dysfunction during the early stages of the pathology. AD and related dementias can be an enormous economic burden for taxpayers, patients, their families, medical systems, and society as a whole (1). According to recent estimates, the total direct medical expenditures associated with AD and related dementias in the United States will increase from $236 billion in 2016 to more than $1 trillion in 2050 due to projected increases in the elderly population (1, 2).

Since a German psychiatrist and neuropathologist, Dr. Alois Alzheimer, introduced the case of memory loss, disorientation, and hallucinations in the patient Auguste D., many follow-up studies have been performed to investigate the pathophysiology of AD. Despite continuing debate, the Aβ hypothesis and tau pathology have become the dominant models of AD pathogenesis in the fields of psychiatry, neurology, and neuroscience. Glenner and colleagues suggested that Aβ, a special amyloid protein accumulated in the brain, could be causative of AD (3). In several studies for more than three decades, researchers have consistently accumulated data and have become increasingly supportive of this theory (4–9). Most researchers have naturally paid attention to Aβ pathology as a promising treatment target for AD. However, this expectation has encountered numerous failures of phase-III clinical trials that aimed to modify AD such as by slowing down or stopping its progression. Consequently, researchers are currently more interested in tau pathology as an alternative therapy target, but more study is needed to determine whether tau pathology could be a major target for disease-modifying treatment.

Although the Aβ hypothesis and tau pathology are important to AD pathology, it could be realistically and pragmatically necessary to discuss the various paradigm shifts to overcome the failures in developing disease-modifying treatments for AD. The literature reviewed below is focused on the value of inquiries on cognitive reserve (CR) and precision medicine (PM) for AD as preventive measures. The attention paid to CR is due to the observation of interindividual variability in cognitive decline without parallel changes to neuropathological processes. In this context, researchers have considered that other factors may affect the path of cognitive function in not yet demented individuals. The concept of PM, also called “personalized medicine” or “individualized medicine,” is rapidly advancing in medical, clinical, and research settings (10). A new paradigm of PM aims to optimize the effectiveness of disease prevention and treatment by taking account of the specific biological compositions of individuals (10, 11).

## Cognitive Reserve

Sister Bernadette (not her real name), a Catholic nun living in the School Sisters of Notre Dame convent, showed no decline in cognitive function and activities of daily life. After her death due to a massive heart attack, an autopsy showed a great spread of AD pathology in her brain. The discordance between the degree of brain pathology and the clinical manifestation in her lifetime suggested that her neocortex was resistant and resilient to Alzheimer’s-related neurodegeneration (12). Katzman and colleagues reported cases of elderly people who had normal cognitive function but were found to have advanced AD pathology in their brains at the time of death (13). Therefore, researchers needed a concept that could explain individual variabilities in cognitive function, activities of daily life, or clinical decline in a manner relative to aging and neurodegenerative disease.

### Definition of CR, Brain Reserve, and Brain Maintenance

#### Cognitive Reserve

Although several studies have defined CRs and related concepts, the terms have been used in conjunction with a common denominator, but in different ways in published studies. A recent whitepaper published by the Reserve, Resilience and Protective Factors PIA Empirical Definitions and Conceptual Frameworks Workgroup defined CR as the adaptability of cognitive processes related to differential susceptibility of cognitive abilities or day-to-day function to brain aging, pathology, or insult (14). This concept concludes that the diversity of an individual’s CR is the result of the interaction between life exposures and genetic factors. Therefore, individuals exhibit differences in adaptation to brain diseases and aging according to their own CR.

#### Brain Reserve

The whitepaper (14) demarcated the concept of a brain reserve, which is distinct from CR. Brain reserve refers to individual variation in the structural characteristics of the brain at any point in time, rather than a macroscopic construct that is not related to verifiable neurobiology or the mechanisms of finer particles. In this context, macroscopic structural characteristics such as total brain volume, volume of a specific neural substrate, or white matter integrity could influence the threshold of the emergence of cognitive impairment.

#### Brain Maintenance

Brain maintenance is conceptually and temporally subdivided from CR, although it is highly relevant to brain reserve. Brain maintenance refers to a decline in the development of age-related changes in the brain and protection against the effects of neuropathology (14). Therefore, brain maintenance influences an individual’s cognitive function for their lifespan through an interaction between lifetime experiences and genetic factors. While brain reserve includes the neurobiological resources of a specific point in time, brain maintenance has the potential to maintain or enhance brain function over time (14).

### Evidence for CR

Most researchers seem to agree that CR is an appropriate concept for describing the interaction between genetic factors and lifetime experiences and consequent phenotypes. Although the definitions of CR are becoming clearer as several studies progress, a number of studies still tend to use the CR definition within various boundaries. The authors will follow the definition of the whitepaper (14) but use a broader definition of concepts when describing evidence for CR.

#### Education, Occupation, Leisure Activities

Individuals with less education were associated with a higher risk of developing dementia compared to those with more education (15, 16). Indeed, education has been widely accepted as one of the proxies of CR. Several studies have presented biological evidence that could support the epidemiologic evidence for CR. Higher education was associated with reduced white matter integrity in the medial temporal lobe areas and association fiber tracts when controlled for age, gender, and dementia severity (17). Higher education is a protective factor against AD and is associated with lower plasma tau levels in patients (18). Through analysis with brain magnetic resonance imaging, the magnitude of the contribution of education is seen as greater than the negative impact of either a neuropathological burden such as white matter hyperintensities or hippocampal atrophy (19).

Occupational attainment acts as a proxy for CR and is associated with a lower risk of AD and a delayed onset of symptoms (20, 21). Moreover, occupational complexity may grant resilience against the negative effects of neuropathology on cognition in people at risk for AD (22). In a fluorodeoxyglucose (^18^F) positron emission tomography (FDG-PET) brain imaging study, Garibotto and colleagues showed an inverse correlation between a reserve index, accounting for educational/occupational level, and metabolism in the posterior cingulate cortex and precuneus in both APOE ε4 carriers and noncarriers. Their results suggested that education and occupation act as proxies for a reserve in epsilon4 carriers, compensating for an unfavorable genetic background (23). However, not all studies have found these relationships; Myung and colleagues found that the protective effect of high occupational attainment against cognitive decline disappeared in the MCI stage (24).

Participation in leisure activities, known to have a protective effect against developing AD, is one of the proxies for CR (25–27). Among leisure activities such as walking for pleasure, visiting friends, reading, playing games, religious activity, physical conditioning, and so on, social, cognitive, and physical leisure activities appear to have protective effects against the risk of dementia (20, 28, 29). Physical activity, particularly aerobic exercise, is protective against age-related gray and white matter loss. Cognitive training of executive functions is associated with an improvement in prefrontal network efficiency (30).

The potential mechanisms of CR are not yet elucidated. However, Engeroff and colleagues showed that regular physical activity might be beneficial for preserving brain plasticity age and was positively associated with brain-derived neurotrophic factor (BDNF) levels in healthy elderly people (31). Ward and colleagues showed that BDNF played an important role in the capacity for building or accessing CR. A significant positive relationship between CR and executive function was identified in BDNF Val homozygotes but was not evident in BDNF Met carriers (32).

#### Other Proxies for CR

Premorbid intellectual function could account for discrepancies in clinical status between MCI and AD patients that have similar levels of neuropathology and comorbid medical diseases (33). Minicolumn thinning of neurons in the cerebral cortex, which is related to cognitive ability, occurs in old age. AD patients with a higher IQ were older and had less pathology at the time of death, which provides the neural evidence for the CR hypotheses (34).

While the results of the prospective cohort study showed that there was no association between bilingualism and the delayed onset of AD, retrospective studies have claimed the opposite (35). However, Perani and colleagues studied brain metabolism, a direct index of synaptic function and density, and neural connectivity to shed light on the effects of bilingualism in AD (36). They observed that bilingual individuals were 5 years older than their monolingual peers on average. Through the metabolic connectivity analyses, they supported the neuroprotective effect of bilingualism by showing an increased connectivity in the executive control and default mode networks in bilingual, compared with monolingual, AD patients. Furthermore, the degree of lifelong bilingualism was significantly correlated to functional modulations in crucial neural networks, suggesting both neural reserve and compensatory mechanisms. They suggested that lifelong bilingualism acts as a powerful CR proxy in dementia and exerts neuroprotective effects against neurodegeneration. However, further studies appear to be needed to assess if bilingualism can be used as a proxy for CR.

Other studies have suggested that learning a foreign language could enhance an individual’s CR. Bubbico and colleagues showed that learning a foreign language significantly improved global cognition, along with increased functional connectivity in the right inferior frontal gyrus, right superior frontal gyrus, and left superior parietal lobule in healthy elderly subjects (37). They suggested that language learning practice could be another important way to enhance and reorganize brain networks.

In the last few years, several large studies investigated lifestyle-related risk factors in people at risk for dementia. The FINGER (Finnish Geriatric Intervention Study to Prevent Cognitive Impairment and Disability) study (38) showed that multidomain intervention had beneficial effects on cognitive functions, especially in executive functioning and processing speed. The control group only received regular health advice, whereas the intervention group received dietary counselling, physical exercise and cognitive training, and vascular risk factor monitoring. These results are in line with the concept of CR described in this paper. Other large studies have shown variable results. The PreDIVA (Prevention of Dementia by Intensive Vascular Care) (39) contradicted the FINGER study by suggesting that multidomain cardiovascular intervention had no positive effect on dementia. In addition, the MAPT (Multidomain Alzheimer Preventive Trial) (40) study included a multidomain intervention group (including integrated cognitive training, physical activity, dietary advice, preventive consultations, and intake of omega-3 polyunsaturated fatty acids (PUFAs)) versus an only multidomain intervention group versus an only omega-3 PUFAs group versus a placebo capsule group. The results presented no significant difference between any of the three intervention groups compared with the placebo control group. Nevertheless, the MAPT study also presented meaningful results in that the multidomain intervention group showed less cognitive decline than groups without multidomain intervention.

## Precision Medicine

PM is a medical approach that recommends preventing and treating diseases based on the unique genetic makeup and lifestyle of an individual. Conceptually and clinically, PM for AD is closely related to CR. The concept of CR includes a heterogeneous phenotype and takes into account that the same pathological findings might not result in the same clinical symptoms. The concept of PM also allows that individuals could be diagnosed with the same disease even if they have different biological makeups, such as genetic, epigenetic, biomarker, phenotypic, lifestyle, and psychosocial characteristics. While the traditional approach to neurodegenerative diseases focuses on brain proteinopathies as homogenous clinicopathological or clinicobiological entities, the new paradigm of PM aims to optimize the effectiveness of disease prevention and treatment by taking into account biological components that could influence the heterogeneity of a disease by considering specific biological factors (11). Up until now, clinicians have usually used a universal treatment strategy by applying the same intervention to a particular disease. While this treatment strategy, the so-called “one-size-fits-all” method, could be very successful for some patients, it may not be effective for others. PM is an innovative approach that embraces individual differences in the genetics, environments, and lifestyles of each individual.

### Genetics in PM

The extensive complexity of the genetics of AD is one of the main causes of clinical and pathological diversity. Since the heritability of AD is estimated to be from 58% to 79% (41, 42), a requisite for prevention and early intervention is to qualitatively and quantitatively obtain a large amount of information about the extensively complex genetic variants in AD. Many studies, including large-scale genome-wide association studies (GWAS), the first round of whole exome sequencing (WES), and whole genome sequencing (WGS), investigated susceptibility loci that were associated with molecular pathways in AD, including the amyloid pathway, immune system, lipid metabolism, and hippocampal synaptic function (10).

Mutations in the amyloid precursor protein (APP, located at chromosome region 21q21.2) (43), presenilin 1, (PSEN1, located at 14q24.3) (44), and presenilin 2 (PSEN2, located at 1q42.13) (45) are well known to cause early onset AD. These genetic mutations have a strong penetration effect on AD pathology.

APOE is the most notable lipoprotein in AD research and is divided into three forms, apoE2, apoE3, and apoE4. The risk of AD is two to three times higher in people with an APOE ε4 allele and about 12 times higher in people with two APOE ε4 alleles (46, 47). The APOE ε4 allele is associated with higher deposits of Aβ in the brain (48). In addition, the APOE genotype may influence the topography of regional atrophy and cortical thinning in AD. Cortical thickness in AD patients was significantly lower in the medial temporal and left parietal regions of the APOE ε4 allele group, and in the medial temporal lobe of the group with two APOE ε4 alleles, compared with controls (49).

Despite significant progress in identifying the underlying genetics, studies have only illuminated the genetic factors underlying the pathophysiology of neurodegenerative diseases. Further genetic studies in the future are expected to clarify the pathogenic mechanisms of AD that could be used for preventing AD and treating AD patients. Since a genetic variant in an individual might contribute a small effect to neuropathology in AD, and a qualitative and quantitative aggregate of susceptibility genes could determine the progress of neurodegeneration, it may be useful to calculate the genetic burden of individuals with a polygenic scoring system. Furthermore, many follow-up studies and expert consensus will be necessary to determine the qualitative and quantitative weights of various genetic information.

### Neuroimaging Data for PM

In their structural MRI research, Tondelli and colleagues reported that the reduced brain volume of the medial temporal lobe such as the hippocampus, amygdala, and entorhinal cortex in cognitively intact individuals is a predictive factor of later cognitive decline (50).

FDG-PET is a nuclear medicine functional imaging technique that is used to observe the cerebral metabolic rate of glucose (CMRglu). Several FDG-PET studies have shown that CMRglu reduction can occur decades before the onset of AD (51). Therefore, individuals with normal cognition have the potential to develop AD if CMRglu reduction is consistently observed in a particular area such as the parieto-temporal areas, posterior cingulate cortex, and medial temporal lobe (51, 52).

In an amyloid PET study, Petersen and colleagues showed that amyloid load *in vivo* was independently associated with a future decline in cognition (53). Elevated amyloid levels were associated with worse cognition, imaging biomarkers, greater clinical decline, and neurodegeneration (54). With ^18^F-flortaucipir and ^18^F-florbetaben positron emission tomography scans, Cho and colleague presented a mutually influential relationship between tau and Aβ deposition. Therefore, investigations of tau and Aβ deposition with PET scans still need to consider the mutual influence between tau and amyloid pathologies.

### Blood Biomarkers

Cerebrospinal fluid biomarker signatures are recognized as useful tools for diagnosing presymptomatic, prodromal, typical, and atypical forms of AD (55). Olsson and colleagues showed that t-tau, P-tau, Aβ42, and NFL levels in the CSF should be used in clinical practice and clinical research for diagnostic purposes (56). However, more research is needed on blood biomarkers that are minimally invasive and relatively inexpensive, unlike the process of obtaining CSF.

Studies of blood biomarkers, however, do not show consistent results for the diagnosis of presymptomatic, MCI, and AD patients. The present limitations to the development of blood biomarkers is that brain-specific proteins must cross the blood-brain barrier and that they are observed at lower concentrations in the blood than in CSF. Nonetheless, high plasma tau was associated with cognitive impairment, brain atrophy, and brain hypometabolism in an Alzheimer’s Disease Neuroimaging Initiative (ADNI) (57). Higher plasma tau was related to lower scores in global cognition, memory, and attention tests and to reduce cortical thickness in AD neural substrates, after adjustments for age, sex, education, and APOE genotype; however, tau levels in MCI were not statistically signiﬁcantly higher than in controls (58).

Several studies have investigated plasma neurofilament light (NFL) as a blood biomarker of neurodegenerative disease. Higher plasma NFL was observed in patients with MCI and AD in comparison with controls. In addition, higher plasma NFL was associated with Aβ pathology in MCI and AD patients. Thus, higher plasma NFL is correlated with poor cognition and atrophy in AD signature regions and with brain hypometabolism (59).

## Discussion

### CR for the Prevention of AD

CR is a widely used term among psychiatrists, neurologist, and neuroscientists who study neurodegenerative diseases. In the psychiatric field, especially regarding posttraumatic stress disorder, adjustment disorder, and depression, resilience is defined as an individual’s ability to adapt to adverse events in life and recover to prestress adaptation levels. In a similar vein, brain resilience is defined as the ability to cope with AD pathology and is measured by a better-than-expected cognitive performance, brain structure, or brain function, despite some level of AD pathology (28). Just as the neurobiology of resilience is under investigation, the neurobiology of CR has also greatly advanced. Indeed, the investigation of genetics, neuroimaging, and epidemiology for CR could be compared to the development of shields that defend against the pathology of neurodegenerative diseases. Stern and colleagues summarized all of the studies that calculated the protective effects of higher CR and found that it reduced the risk of developing dementia by 46% (20). The studies mentioned above suggest a significant mechanism of higher CR for the preservation of cognitive function, which is associated with protection against disease-related cognitive decline. This paper refers to several proxies for CR, such as education, occupational attainment, leisure activities, premorbid intellectual function, and bilingualism. Among the proxies, some are capable of increasing CR by promoting educational and occupational opportunities through individual effort and policy-based approaches. Public authorities must promote many education and occupational attainment opportunities for young people. These policies have to encompass lifelong education (home schooling, adult education, job training, learning a foreign language, etc.) and social, physical, and cognitive leisure activities for the elderly.

There are also interesting studies in a different context. Once symptoms of dementia appear, individuals with a higher reserve (e.g., greater education, occupational attainment, or more leisure activities) are hypothesized to be associated with a more rapid cognitive decline and died sooner than those with lower reserves (60–63). Since individuals with higher CR could be resistant and resilient to more neuropathology, higher levels of CR are also hypothesized to be associated with a faster rate of cognitive decline after the neuropathology passes over a certain threshold and emerges as cognitive decline (60). Although individuals with higher levels of CR are resistant and resilient enough to withstand advanced neuropathology, after crossing the critical threshold, they have little brain reserves left to endure neurodegeneration. Nevertheless, it cannot be worthless to elevate CR levels. Some estimates indicate that delaying the onset of dementia by only 5 years would result in a 50% reduction in dementia prevalence (64).

### PM for the Prevention of AD

Conceptually, PM is a model that supports integrated research and clinical approaches. Hampel and colleagues presented the framework of the Alzheimer Precision Medicine Initiative (APMI) (65). The contents of this model are as follows: (1) collection of big and deep data consisting of biomolecular, imaging, literature, and clinical data through research and clinical practice, (2) processing heterogeneous multidimensional big and deep data through standardization, management, integration, and analysis, and (3) developing an “actionable” model that predicts the trajectory of individualized, patient-centric detection, or treatment within a P4 (predictive, preventive, personalized, and participatory) implementation strategy. An integrated approach such as this model could be a valuable paradigm shift for researchers and clinicians trying to overcome the “one-size-fits-all” treatment that has now revealed its limitations. In the field of oncology, PM seems to have made significant progress in the standard of care by incorporating genetic information and biomarkers. However, PM for AD might need to be investigated from a different and more complex point of view than the field of oncology. This paper has discussed CR as a protective factor against the pathology of neurodegenerative diseases. The authors suggest that researchers and clinicians should consider the CR of an individual at risk of AD whenever they use PM to establish a prevention and treatment plan. In other words, although risk assessment with PM may be the same between individuals, the patient with a lower CR may need more aggressive prevention and treatment plans.

The authors suggest that a system of integrating and interpreting results from research with enormous biological implications must be established in order to bring about a successful paradigm shift from traditional medicine to PM. In this context, data science, which is a field of study dedicated to the principled extraction of knowledge from complex data (66), will be widely applied to the field of PM. Data science already plays a significant role in many human activities and in the world of science. This field is supported by the remarkable and super high-speed development of artificial intelligence and machine learning, despite positive and negative opinions about its development. The development of traditional research areas such as genetics, neuroimaging and biomarkers, and the innovation of data science, which encompasses and incorporates research from these areas, will certainly make a significant contribution to the personalization of prevention and treatment strategies using PM.

## Conclusions

We suggest that research into CR and PM could be a key to overcoming the limitations of traditional approaches in the prevention and treatment of AD.

## Author Contributions

CH was responsible for conception and design as well as initial drafting of the manuscript. All authors (CH and CL) were responsible for revising the manuscript critically for important intellectual content of the version of the manuscript to be published. All authors read and approved the final manuscript.

## Funding

This research was supported by the Ministry of Trade, Industry and Energy (MOTIE, Korea) under Industrial Technology Innovation Program No10062378.

## Conflict of Interest

The authors declare that the research was conducted in the absence of any commercial or financial relationships that could be construed as a potential conflict of interest.
