# Corrigendum: A Brief Review of Paradigm Shifts in Prevention of Alzheimer's Disease: From Cognitive Reserve to Precision Medicine

**DOI:** 10.3389/fpsyt.2019.00900

**Published:** 2019-12-17

**Authors:** Changtae Hahn, Chang Uk Lee

**Affiliations:** ^1^ Department of Psychiatry, Deajeon Saint Mary's Hospital, College of Medicine, The Catholic University of Korea, Seoul, South Korea; ^2^ Department of Psychiatry, Seoul Saint Mary's Hospital, College of Medicine, The Catholic University of Korea, Seoul, South Korea

**Keywords:** Alzheimer's disease, cognitive reserve, precision medicine, prevention, aging, biomarkers

In the original article, there was an error regarding the corresponding author and the correspondence address provided. Instead of “Changtae Hahn, jihan@catholic.ac.kr” the correspondence should be addressed to “Chang Uk Lee, jihan@catholic.ac.kr”.

The authors apologize for this error and state that this does not change the scientific conclusions of the article in any way. The original article has been updated.

